# Regional socioeconomic and environmental correlates of mushroom poisoning: an ecological panel study in Sichuan Province, China

**DOI:** 10.3389/fpubh.2026.1866167

**Published:** 2026-07-21

**Authors:** Ruyue Hu, Wen Chen, Li Lin, Yi Xu, Yu Zhang, Yang Song

**Affiliations:** Sichuan Center for Disease Control and Prevention, Chengdu, China

**Keywords:** associated factors, ecological study, mushroom poisoning, panel regression, social determinants of health framework, surveillance

## Abstract

Mushroom poisoning is a significant foodborne disease in China, with southwestern regions being high-risk areas. These incidents place ongoing pressure on primary healthcare facilities and the public health surveillance system. This study aimed to examine area-level factors associated with reported mushroom poisoning case incidence using aggregated prefecture-level data in Sichuan Province. This ecological panel study used surveillance data from the *National Foodborne Disease Outbreak Surveillance System* in Sichuan Province from 2019 to 2024. The unit of analysis was prefecture-level cities, and all variables were aggregated at the regional level. Potential determinants were selected based on the Social Determinants of Health framework. Panel regression models were used to estimate associations between area-level variables and reported mushroom poisoning case incidence. From 2019 to 2024, Sichuan Province reported 1,029 mushroom poisoning incidents involving 3,551 cases. The average annual reported incidence rate was 7.07 per million people, showing a fluctuating upward trend over the study period. In the multivariable analysis, per capita GDP (*β* = 0.109, 95% CI: 0.016 to 0.202) was positively associated with the reported mushroom poisoning case incidence, while the climate index (*β* = −0.072, 95% CI: −0.118 to −0.026) and urbanization rate (*β* = −0.140, 95% CI: −0.224 to −0.056) were negatively associated with incidence. The medical staff allocation index (*β* = 0.069, 95% CI: 0.010 to 0.129) and education level index (*β* = 0.064, 95% CI: 0.010 to 0.119) were positively associated with the reported incidence rate. Between 2019 and 2024, the reported mushroom poisoning case incidence in Sichuan Province exhibited an increasing trend with substantial regional variation. Regional differences in reported incidence may be associated with a combination of socioeconomic conditions, environmental context, and healthcare system characteristics. Strengthening surveillance capacity, improving mushroom species identification, and implementing region-specific health education and risk communication strategies may help reduce the burden of mushroom poisoning.

## 1 Introduction

Mushroom poisoning is one of the long-standing significant foodborne diseases in China (1), characterized by rapid onset, fast progression, and potentially severe outcomes, including acute liver failure and death. Although its occurrence is geographically concentrated, particularly in southwestern China, mushroom poisoning represents a typical form of foodborne disease driven by the interaction between environmental conditions and human behaviors.

In recent years, due to ecological changes, increased population mobility, and more frequent tourism activities, mushroom poisoning incidents have shown a high incidence and regional clustering, reflecting epidemic characteristics (2). The southwestern regions of China, such as Sichuan, Yunnan, Guizhou, and Chongqing, are considered high-risk areas due to their humid climate, complex vegetation, and long-standing dietary practices involving wild mushroom consumption. The incidence is strongly seasonal, with peaks observed during summer and autumn months (3, 4). These characteristics highlight the role of environmental exposure, behavioral patterns, and regional context in shaping disease risk, and place sustained pressure on primary healthcare systems and public health surveillance capacity.

The Chinese Center for Disease Control and Prevention (CDC) releases a national report on mushroom poisoning incidents annually. The latest annual report indicates that China reported 599 mushroom poisoning incidents, with over 1,400 cases of poisoning and 13 deaths, resulting in a case fatality rate of 0.87% (1) in 2024. This provides an important basis for understanding the national trends in mushroom poisoning. However, the monitoring results mainly rely on the *National Public Health Emergency Surveillance System*, which typically only reports mass incidents (usually involving ≥30 people) of mushroom poisoning. As a result, many sporadic cases and small-scale household cluster incidents are not systematically captured, leading to an underestimation of the true disease burden of mushroom poisoning. China National Center for Food Safety Risk Assessment (5) (CFSA) further analyzed mushroom poisoning incidents from 2010 to 2020 based on the *National Foodborne Disease Outbreak Surveillance System*, reporting more than 10,000 incidents nationwide, with over 38,000 cases of poisoning and nearly 800 deaths. The overall trend has been steadily increasing, with the highest incidence rates found in the southwestern and central regions. More than 80% of outbreaks were related to family food preparation. However, beyond surveillance-based descriptions, the factors underlying regional variation in reported mushroom poisoning case incidence remain poorly understood.

Previous studies on mushroom poisoning have mainly focused on three aspects. First, epidemiological studies have described the temporal and spatial patterns of poisoning incidents (6, 7). Second, researches on pathogenic species have examined the composition and distribution of toxic mushroom varieties (8, 9). Third, clinical studies have investigated the characteristics and outcomes of poisoning cases (10–12). However, these studies are often limited to descriptive analyses, single regions, or short time periods, and typically focus on isolated factors. Consequently, there remains a lack of comprehensive quantitative analyses that simultaneously consider multiple determinants across regions and over time, particularly with respect to socioeconomic conditions, environmental factors, and health system characteristics.

Previous research has suggested that multiple contextual factors may be associated with the occurrence of mushroom poisoning and other environmentally related foodborne diseases. Socioeconomic conditions, such as income level, have been linked to differences in food acquisition patterns (13, 14), with lower-income populations more likely to engage in foraging behaviors that increase exposure risk (15). Educational attainment (16) has been associated with variations in risk awareness and the ability to identify toxic species. Environmental factors (17, 18), particularly climatic conditions such as temperature and precipitation, might influence the growth and distribution of wild mushrooms, thereby shaping exposure risk. In addition, health system capacity may affect the detection, reporting, and management of poisoning cases, as areas with greater healthcare resources may show higher reported incidence due to improved surveillance and diagnostic capacity (19). However, existing studies are often limited to specific regions or individual factors, and there is a lack of integrated analyses examining these determinants simultaneously across regions and over time.

The Social Determinants of Health (SDH) framework conceptualizes health outcomes as arising from the interaction of socioeconomic conditions, environmental context, and health system capacity (20). Within this framework, mushroom poisoning can be understood as a context-dependent foodborne disease, the risk of which may be associated with factors such as economic conditions, educational attainment, healthcare resource availability, and climatic environment (21). These factors may shape patterns of exposure, risk awareness, and case detection at the regional level. However, empirical evidence examining these associations in China remains limited, particularly regarding the simultaneous integration of multiple determinants across regions and over time.

Based on this, we utilized data from the Sichuan Province Foodborne Disease Surveillance Platform from 2019 to 2024 to examine regional variation in reported mushroom poisoning case incidence based on outbreak surveillance data. Guided by the SDH framework, we conducted an ecological panel analysis using prefecture-level data to assess associations between socioeconomic conditions, environmental factors, and health system characteristics and reported mushroom poisoning case incidence based on outbreak surveillance data across regions and over time. The aim is to provide scientific evidence for the precise prevention and control of mushroom poisoning and the optimization of resource allocation.

## 2 Materials and methods

### 2.1 Study design and data sources

#### 2.1.1 Study design

This study was a longitudinal ecological panel study based on annual prefecture-level data from 21 cities/prefectures in Sichuan Province from 2019 to 2024. The unit of analysis was the prefecture-level region-year observation. All outcome and explanatory variables were aggregated at the regional level.

#### 2.1.2 Mushroom poisoning incidents and cases

The data for this study was obtained from the Sichuan Province mushroom poisoning surveillance data in the *National Foodborne Disease Outbreak Surveillance System* from 2019 to 2024. For each prefecture-level city/region and calendar year, aggregated data were extracted on the annual number of mushroom poisoning incidents and the annual number of mushroom poisoning cases. These annual prefecture-level counts were linked with regional population data to calculate the annual reported incidence rate of mushroom poisoning cases per million population. According to the relevant regulations in the *National Foodborne Disease Surveillance Manual*, foodborne disease outbreaks are defined as incidents where at least two individuals are affected or at least one death occurs. Accordingly, in this study, mushroom poisoning incidents with at least two cases of poisoning or any fatalities were classified as mushroom poisoning outbreak events.

The outcome of this study should be interpreted as the reported incidence of outbreak-associated mushroom poisoning cases captured by the surveillance system, rather than the true population incidence of all mushroom poisoning cases. Sporadic non-fatal cases that did not meet the outbreak reporting definition may not have been systematically captured.

#### 2.1.3 Data sources for potential determinants

Data on potential influencing factors mainly came from the *Sichuan Provincial Statistical Yearbook (2020–2025).* The following indicators for each city/prefecture in Sichuan Province from 2019 to 2024 were obtained: per capita Gross Domestic Product (GDP, RMB), per capita disposable income (RMB), annual precipitation (mm), annual average temperature (°C), urbanization rate (%), proportion of population with high school education or above (%), number of hospital beds per 1,000 people, number of medical institutions (units), and number of healthcare personnel (persons). All variables were available for each prefecture-year observation, and no imputation or substitution of missing values was performed.

#### 2.1.4 Indicator calculation and data processing

The annual reported incidence rate was calculated as the number of reported mushroom poisoning cases divided by the corresponding resident population expressed in units of 10,000 persons. A climate index was constructed by standardizing annual precipitation and average temperature and then summing the standardized values to capture overall climatic conditions at the regional level. The medical staff allocation index was defined as the ratio of healthcare personnel to the number of medical institutions in each prefecture.

To reduce skewness and stabilize variance, variables bounded between 0 and 1 in this dataset, including the reported mushroom poisoning case incidence, urbanization rate, and education level index, were transformed using the arcsine square-root transformation. For descriptive presentation, the reported mushroom poisoning case incidence was also expressed as cases per million population. Continuous variables with different units and scales, including per capita GDP, per capita disposable income, climate index, hospital beds per 1,000 population, and the medical staff allocation index, were standardized prior to regression analysis to improve comparability and model stability.

### 2.2 Theoretical framework

This study was guided by the Social Determinants of Health (SDH) framework proposed by the World Health Organization (WHO), which conceptualizes health outcomes as arising from the interaction of structural determinants, intermediary determinants, and health system factors (20). In this study, the SDH framework was used to inform both the selection and classification of explanatory variables. Structural determinants included indicators reflecting socioeconomic conditions, such as per capita GDP, per capita disposable income, urbanization rate, and education level. However, per capita GDP and per capita disposable income were both considered indicators of regional economic conditions. Due to their conceptual overlap and high correlation, including both variables in the same model may introduce multicollinearity. Therefore, per capita GDP was selected as the primary indicator of regional economic development in the main analysis, while urbanization rate and education level were retained as additional structural determinants within the SDH framework.

Intermediary determinants were represented by environmental conditions, captured through the climate index. Health system determinants included indicators of healthcare resource availability, such as hospital beds per 1,000 population and the medical staff allocation index. All variables were specified *a priori* based on this conceptual framework and were included in the multivariable analyses to examine their associations with reported mushroom poisoning case incidence based on outbreak surveillance data.

Additional methodological details are provided in the Supplementary material.

### 2.3 Panel regression model

This study employs a panel regression model to examine associations between area-level determinants and reported mushroom poisoning case incidence based on outbreak surveillance data at the prefecture level in Sichuan Province. The panel data model allows adjustment for unobserved time-invariant heterogeneity across prefecture-level regions by combining cross-sectional and longitudinal variation. The dependent variable was arcsine square-root transformation annual reported mushroom poisoning incidence rate. Explanatory variables included socioeconomic, environmental, and health system indicators defined within the SDH framework.

A pooled panel model was first estimated as the baseline specification. Fixed-effects and random-effects models were then fitted to account for unobserved prefecture-specific effects. In the fixed-effects model, these region-specific effects were allowed to correlate with the explanatory variables, whereas the random-effects model assumed no such correlation. Model selection was based on the F-test and Hausman test. A significant F-test indicated the presence of prefecture-specific effects, favouring the use of fixed-effects or random-effects models over the pooled model. The Hausman test was then used to compare the fixed-effects and random-effects specifications, with a significant result supporting the fixed-effects model and a non-significant result supporting the random-effects model. When heteroscedasticity was identified during model diagnostics, statistical inference was based on heteroscedasticity-robust standard errors clustered at the prefecture level.

Detailed model specifications, sensitivity analyses and model diagnostics (including multicollinearity, heteroscedasticity, and autocorrelation tests) are provided in Supplementary material.

### 2.4 Ethics statement

This study analyzed anonymized routine public health surveillance data together with aggregated data obtained from publicly available statistical sources. No identifiable personal information was involved. According to institutional policy, ethical approval was not required because the study involved secondary analysis of anonymized surveillance data collected for routine public health purposes.

### 2.5 Statistical analysis

Data processing and statistical analysis were conducted using Excel 2021, R 4.5.0, and Python 3.12. Excel was used to clean and organize the raw data. Panel data regression analysis was performed using the *plm* package in R. Line charts and incidence heatmaps were done using the *matplotlib* module in Python. Based on the SDH framework and prior evidence, all prespecified socioeconomic, environmental, and health system variables were included in the multivariable panel regression model regardless of their statistical significance in univariable analyses. Prior to model fitting, collinearity among explanatory variables was assessed. Model selection was determined based on the results of the F-test and Hausman test, and parameter estimation and result interpretation were subsequently carried out based on these decisions. Regression coefficients with 95% confidence intervals (CIs) were reported to describe the direction, magnitude, and uncertainty of associations. All statistical tests were two-sided, with a significance level set at *α* = 0.05.

## 3 Results

### 3.1 Descriptive characteristics

Descriptive statistics of the study variables are presented in Table 1. The panel dataset included 21 prefecture-level regions over six years (2019–2024), resulting in 126 prefecture-year observations. The average reported mushroom poisoning incidence was 9.63 per million population, with substantial variation across regions and years. Socioeconomic, environmental, and healthcare-related variables also showed considerable heterogeneity across prefectures.

**Table 1:** Descriptive statistics of study variables across prefecture-year observations in Sichuan Province, 2019–2024.

Variable	Mean	SD	Min	Max
Reported mushroom poisoning case incidence (per million population)	9.63	14.32	0.00	95.41
Per capita GDP (thousand yuan)	58.29	19.62	22.72	114.32
Medical staff allocation index	10.13	3.74	3.59	22.50
Climate index	0	1.38	−5.12	1.62
Education level (%)	8.86	6.38	1.95	26.32
Urbanization rate (%)	51.88	9.99	30.10	80.81
Hospital beds per 1,000 population	8.91	4.53	1.09	30.21

### 3.2 Mushroom poisoning incidents and cases in Sichuan Province (2019–2024)

From 2019 to 2024, a total of 1,029 mushroom poisoning incidents and 3,551 cases were reported in Sichuan Province. The overall reported mushroom poisoning case incidence showed a fluctuating upward trend during the study period. The highest number of incidents was observed in 2024 (344 incidents), while the lowest numbers were recorded in 2021 and 2022 (both 108 incidents). Since 2022, both the number of incidents and cases have increased, with more pronounced increases observed during 2023–2024.

The annual average reported mushroom poisoning case incidence in Sichuan Province was 7.07 per million population. The highest number of cases occurred in 2024 (1,153 cases), corresponding to an annual reported case incidence rate of 13.78 per million population, while the lowest number of cases occurred in 2022 (376 cases, 4.49 per million population).

**Figure 1:**
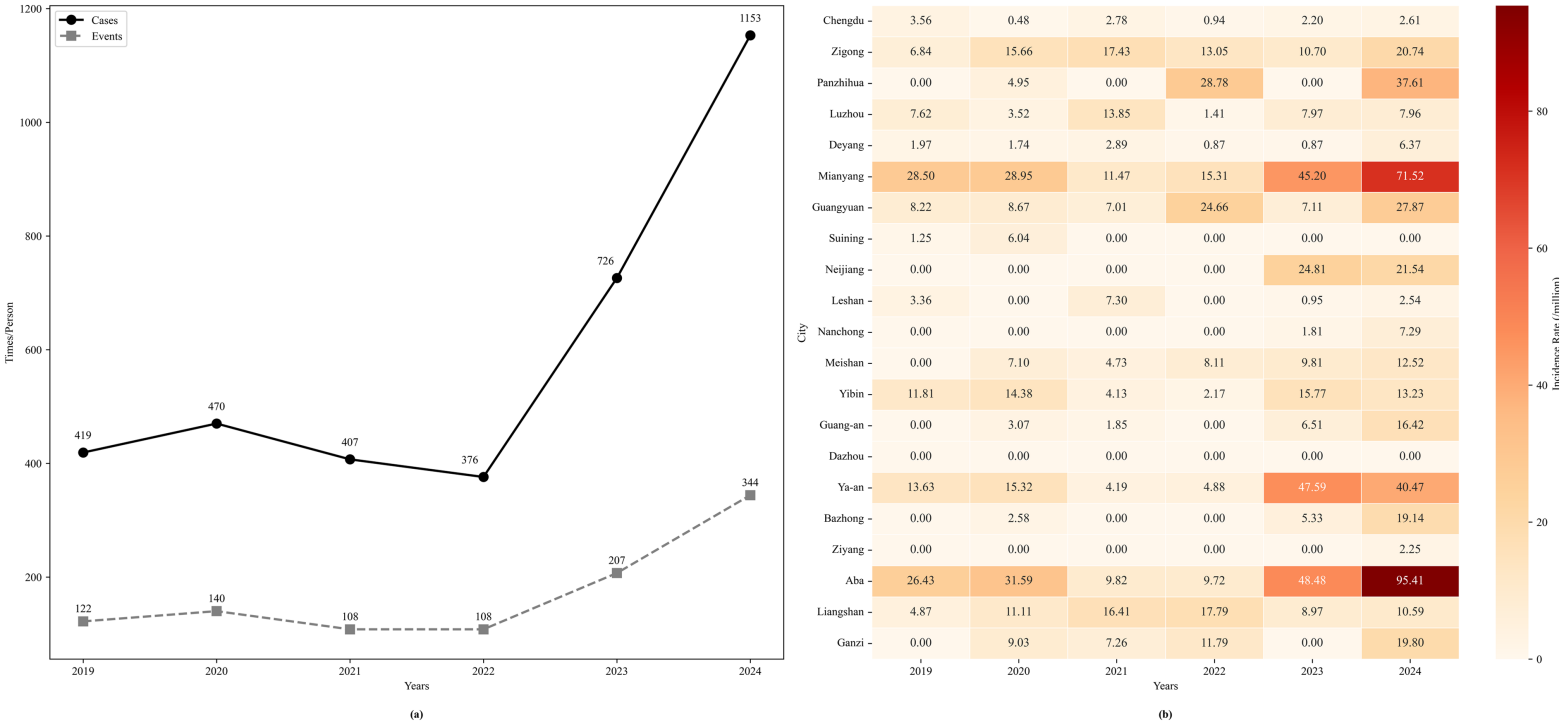
Temporal trends and spatial distribution of reported mushroom poisoning case incidence based on outbreak surveillance data in Sichuan Province, 2019–2024. **(a)** Annual trend of mushroom poisoning incidents in Sichuan Province, 2019-2024. **(b)** Spatio-temporal distribution heatmap of mushroom poisoning incidents in Sichuan Province, 2019-2024.

Regionally, the reported mushroom poisoning case incidence also varied across cities and prefectures. In 2024, Aba Prefecture had the highest reported incidence at 95.41 per million population, followed by Mianyang City (71.52 per million population). No mushroom poisoning incidents were recorded in Dazhou City during the study period, corresponding to a reported incidence rate of zero (Figure 1).

### 3.3 Model selection

After confirming that there was no problematic multicollinearity among the explanatory variables, model selection was based on the results of the F-test and Hausman test. The F-test indicated the presence of significant prefecture-specific effects (*F* = 4.173, *p* < 0.001), suggesting that a panel model was more appropriate than the pooled model. Further Hausman test results indicated that the null hypothesis was not rejected (*χ^2^* = 2.074, *p* = 0.722), suggesting that the random-effects model is more appropriate. Therefore, the random-effects model was ultimately selected for analysis.

Model diagnostic results, including multicollinearity, heteroscedasticity, serial correlation, residual diagnostics, and model goodness-of-fit, are presented in Supplementary Table S1 and Supplementary Figure S1. Sensitivity analyses using a random-effects negative binomial model yielded broadly consistent results with the primary panel regression model (Supplementary Table S3).

### 3.4 Multivariate analysis

The results of the multivariable random-effects panel regression analysis are presented in Table 2. Specifically, per capita GDP was positively associated with the reported case incidence (*β* = 0.109, 95% CI: 0.016 to 0.202). The climate index (*β* = −0.072, 95% CI: −0.118 to −0.026) and urbanization rate (*β* = −0.140, 95% CI: −0.224 to −0.056) were negatively associated with incidence. The medical staff allocation index (*β* = 0.069, 95% CI: 0.010 to 0.129) and education level index (*β* = 0.064, 95% CI: 0.010 to 0.119) were positively associated with the reported incidence rate. No significant association was observed for hospital beds per 1,000 population.

**Table 2:** Multivariable random-effects panel regression analysis of reported mushroom poisoning case incidence in Sichuan Province.

Variable	*β*	SE	t	*p*	95% CI
Per capita GDP	0.109	0.047	2.304	0.023	(0.016, 0.202)
Medical staff allocation index	0.069	0.031	2.282	0.024	(0.010, 0.129)
Climate index	−0.072	0.023	−3.082	0.003	(−0.118, −0.026)
Education level index	0.064	0.028	2.307	0.023	(0.010, 0.119)
Urbanization rate	−0.14	0.043	−3.273	0.001	(−0.224, −0.056)
Hospital beds per 1,000 people	−0.025	0.014	−1.735	0.085	(−0.053, 0.003)

GDP means Gross Domestic Product.

The main findings were broadly consistent in direction across alternative model specifications and sensitivity analyses (see Supplementary Table S2). Additional adjustment for calendar year yielded broadly consistent associations with the primary analysis (see Supplementary Table S4). Sensitivity analyses replacing the composite climate index with temperature and precipitation separately also yielded generally consistent findings (see Supplementary Table S5).

## 4 Discussion

Mushroom poisoning remains an important public health concern in southwestern China, characterized by pronounced regional clustering and substantial health burden, placing sustained pressure on primary healthcare and public health systems (22). This study, based on surveillance data from 21 cities/prefectures in Sichuan Province from 2019 to 2024, applied the SDH framework and constructed a panel regression model to examine regional variation in reported mushroom poisoning case incidence and its associated factors. Consistent with the descriptive findings, reported mushroom poisoning case incidence in Sichuan Province showed an overall increasing trend with substantial temporal fluctuations during the study period. A total of 1,029 mushroom poisoning incidents and 3,551 cases were reported, corresponding to an annual average reported mushroom poisoning case incidence of 7.07 per million population. Substantial regional variation in reported mushroom poisoning case incidence was observed across prefectures. Additionally, per capita GDP, the medical staff allocation index, and education level were positively associated with regional variation in the reported mushroom poisoning case incidence, while the climate index and urbanization rate were negatively associated with reported case incidence.

The observed increase in reported mushroom poisoning case incidence over time, particularly in 2023–2024, may reflect a combination of changes in exposure patterns and improvements in surveillance capacity. Economic growth and the expansion of tourism in Sichuan may have created regional contexts associated with increased opportunities for wild mushroom exposure (23). Meanwhile, ongoing strengthening of the foodborne disease surveillance system, including improved case detection and reporting at the primary healthcare level, may also have contributed to the observed rise in reported case incidence (5). In addition, the standardization of public health emergency responses and the establishment of technical requirements for mushroom species identification by local CDCs and national surveillance systems may also have improved case ascertainment and reporting completeness, thereby contributing to higher reported incidence (1, 9). Taken together, these considerations suggest that the increasing trend in reported incidence may partly reflect improved surveillance and reporting capacity, rather than solely a true increase in underlying risk of mushroom poisoning.

Consistent with these findings, substantial regional variation was observed, with Aba Prefecture and Mianyang City exhibiting reported case incidence higher than the provincial average in 2024. These areas are characterized by plateau or mountainous terrain, humid climatic conditions, and complex vegetation, which provide favorable environments for the growth of toxic wild mushrooms (24). Combined with regional dietary traditions and tourism activities (23), these ecological conditions may create a regional context associated with greater opportunities for human exposure to wild mushrooms, as morphologically similar toxic and edible species may complicate accurate species identification (25). However, given the ecological study design, these associations should be interpreted as regional-level correlations rather than evidence of individual-level causal mechanisms, and may also reflect differences in reporting practices and healthcare access across regions.

This study found that per capita GDP was positively associated with reported mushroom poisoning case incidence, suggesting that regions with higher levels of economic development may report higher reported case incidence. This pattern may reflect regional characteristics associated with economic development, including greater tourism activity and differences in surveillance capacity, healthcare accessibility, and reporting completeness (26, 27). These factors may partly explain higher reported incidence rather than necessarily indicating a higher underlying risk of mushroom poisoning. The urbanization rate was negatively associated with reported case incidence, suggesting that more urbanized regions may have environmental and socioeconomic characteristics associated with reduced opportunities for human exposure to wild mushrooms. This association may reflect differences in environmental context, food supply systems, healthcare accessibility, and reporting completeness between urban and less urbanized regions, although these potential mechanisms cannot be directly evaluated using aggregated ecological data.

The climate index, a composite measure of temperature and precipitation, was negatively associated with the reported mushroom poisoning case incidence. This finding differs from those reported in previous studies (2, 28, 29). This discrepancy may partly reflect differences in study design, analytical approaches, and the use of a composite climate index rather than separate climatic variables. The observed negative association may also reflect the complex interactions between regional climatic conditions and reported mushroom poisoning case incidence (30), which could involve differences in environmental context, exposure opportunities, and surveillance patterns (24). However, the climate index may not fully capture the independent effects of temperature and precipitation on reported mushroom poisoning case incidence. Therefore, this finding should be interpreted cautiously. Future studies incorporating individual climatic variables or more refined environmental indicators may provide a clearer understanding of their respective contributions to mushroom poisoning risk. Nevertheless, sensitivity analyses using temperature and precipitation separately yielded generally consistent findings, supporting the consistency of the observed association.

The medical staff allocation index and education level were positively associated with reported mushroom poisoning case incidence. These patterns may reflect differences in healthcare accessibility, surveillance capacity, and reporting practices across regions (31, 32), rather than direct effects on mushroom poisoning risk itself. The positive association observed for the medical staff allocation index may instead reflect that areas with stronger healthcare systems achieve more complete case detection and reporting, resulting in higher reported incidence (33). Similarly, higher educational attainment may serve as a proxy for broader socioeconomic development rather than acting as an independent determinant of reported mushroom poisoning case incidence (34). However, given the ecological study design, these interpretations should be considered exploratory rather than evidence of direct causal relationships.

Overall, these findings suggest that variation in reported mushroom poisoning case incidence may reflect a combination of structural socioeconomic factors, environmental context, and healthcare system characteristics, consistent with the SDH framework (35).

### 4.1 Study limitations

This study has some limitations. First, the ecological study design precludes causal inference, and the observed regional-level associations may be affected by unmeasured confounding and population heterogeneity across regions. Second, the use of passive surveillance data may be subject to underreporting and reporting bias. Moreover, the surveillance system primarily captures outbreak-associated incidents, so sporadic mushroom poisoning cases may not be systematically recorded, which could influence the observed patterns of reported incidence and regional comparisons. Third, the relatively short study period restricts the assessment of long-term temporal changes and delayed effects. Longer-term surveillance data and more advanced longitudinal modelling approaches may provide additional insights. Fourth, the composite climate index was designed to represent overall regional climatic conditions and may not fully capture the independent effects of temperature and precipitation. Future studies incorporating more detailed climatic indicators may better characterize environmental influences on mushroom poisoning. Finally, the surveillance system did not routinely collect information on toxicological syndromes. Future studies incorporating clinical, toxicological, and pathological data are needed to explore syndrome-specific epidemiological patterns and disease burden (36, 37).

## 5 Conclusion

From 2019 to 2024, the number of mushroom poisoning incidents and cases in Sichuan Province showed an overall increasing trend, with substantial regional variation. In the multivariable analysis, per capita GDP, climate index, urbanization rate, the medical staff allocation index, and education level were associated with the reported incidence of mushroom poisoning. These findings suggest that regional differences in reported incidence may be related to a combination of socioeconomic conditions, environmental context, and healthcare system characteristics. Strengthening surveillance capacity and improving mushroom species identification remain important priorities. In addition, region-specific health education and risk communication strategies, tailored to local socioeconomic and environmental contexts, may help reduce the burden of mushroom poisoning in Sichuan Province.

## Data Availability

The original contributions presented in the study are included in the article/Supplementary material, further inquiries can be directed to the corresponding author.
