# Supplementary material for: Regional socioeconomic and environmental correlates of mushroom poisoning: an ecological panel study in Sichuan Province, China
Source: Front Public Health. 2026 Jul 21;14:1866167. doi: 10.3389/fpubh.2026.1866167 (PMC13433438; doi:10.3389/fpubh.2026.1866167)
Supplement: Supplementary file 1 [file Supplementary_file_1.DOCX]

# Supplementary Material

**Supplemental Methods**

1.Theoretical Framework and Variable Classification

This study was informed by the World Health Organization’s Social Determinants of Health (SDH) framework, which conceptualizes health outcomes as the result of interactions among structural determinants, intermediary determinants, and health system determinants. This framework was used to guide the classification of explanatory variables included in the panel regression analysis.

Structural determinants represent macro-level socioeconomic conditions that shape social stratification and resource distribution. In this study, per capita GDP, the urbanization rate, and the education level index were classified as structural determinants.

Intermediary determinants reflect environmental and contextual conditions through which socioeconomic structures influence health risks. A composite climate index was constructed to capture regional climatic conditions potentially affecting the growth of poisonous mushrooms and population exposure.

Health system determinants describe the capacity of healthcare services for disease prevention, diagnosis, and treatment. Indicators including hospital beds per 1,000 people and the medical staff allocation index were used to represent regional healthcare resource availability.

2.Panel Regression Model Specification

Panel regression models were used to examine factors associated with mushroom poisoning incidence at the prefecture level in Sichuan Province. The panel structure incorporated both cross-sectional (prefecture-level) and longitudinal (yearly) variation, allowing control for unobserved time-invariant heterogeneity across prefectures.

The dependent variable was the reported mushroom poisoning case incidence, transformed using a square root inverse sine transformation to stabilize variance. Explanatory variables included per capita GDP, climate index, hospital beds per 1,000 population, medical staff allocation index, as well as the urbanization rate and education level index, both of which were also square root inverse sine-transformed.

An initial pooled panel regression model was estimated as the baseline specification. Fixed-effects and random-effects models were subsequently fitted to account for unobserved prefecture-specific effects.

Model selection followed a two-step procedure. First, an F-test was conducted to assess the presence of significant prefecture-specific effects. Second, a *Hausman* test was applied to compare fixed-effects and random-effects specifications. A non-significant *Hausman* test supported the use of the random-effects model.

3. Model Diagnostics

To further evaluate the assumptions and performance of the primary random-effects panel regression model, model diagnostic analyses were conducted. Multicollinearity was assessed using variance inflation factors (VIFs). Heteroscedasticity was evaluated using the Breusch–Pagan test, and serial correlation was assessed using the Breusch–Godfrey test for panel models. Residual diagnostics were performed by visual inspection of the residuals-versus-fitted plot. Model goodness-of-fit was evaluated using the Akaike information criterion (AIC) and Bayesian information criterion (BIC).

4. Sensitivity Analyses

To assess the robustness of the main findings, additional analyses were conducted using alternative model specifications. Specifically, one-period lagged values of selected explanatory variables were introduced to explore potential temporal ordering and assess the stability of the estimated associations.

The sensitivity models were estimated using the same panel structure and estimation strategy as in the main analysis. All other model settings remained unchanged.

As an additional sensitivity analysis, we fitted a random-effects negative binomial regression model using the annual reported mushroom poisoning case counts as the outcome and the logarithm of the prefecture-level population as an offset to account for differences in population size. The model was used to accommodate the count nature of the surveillance data and potential overdispersion. The consistency in the direction and magnitude of the estimated associations between this model and the primary panel regression model was evaluated to assess the robustness of the primary findings. Additionally, calendar year was included as a categorical variable in the random-effects panel regression model to evaluate the potential influence of secular temporal trends.

To evaluate whether the observed association depended on the construction of the composite climate index, additional sensitivity analyses were conducted by replacing the climate index with standardized temperature and standardized precipitation separately.

**Supplemental results**

1. Model diagnostics results

All VIF values were below 5, indicating no evidence of problematic multicollinearity(**Table S1**). The Breusch-Godfrey test showed no evidence of serial correlation(*χ²=*5.322*,P=*0.503). The Breusch-Pagan test suggested heteroscedasticity(*χ²=*27.585*,P<* 0.001). Residual plots did not reveal substantial systematic deviations(**Figure.S1**). The AIC and BIC of the primary random-effects panel regression model were 1000.61 and 1028.98, respectively. Taken together, these diagnostic results, along with the supplementary sensitivity analyses, increase confidence in the reliability of the primary findings.

**Table S1 Variance inflation factors for explanatory variables in the primary panel regression model**

| Variable | VIF |
| --- | --- |
| Per capita GDP | 4.1 |
| Medical Staff Allocation Index | 3.24 |
| Climate Index | 1.38 |
| Education Level Index | 1.33 |
| Urbanization Rate | 4.57 |
| Hospital Beds per 1,000 People | 1.22 |

Note: All VIF values were below 5,indicating no evidence of problematic multicollinearity.

Figure.S1 Residuals-versus-fitted plot for the primary random-effects panel regression model

2. **Sensitivity analysis**

2.1: One-period lag model

Table S2 presents the results of the sensitivity analysis using one-period lagged explanatory variables to address potential reverse causality. Overall, the direction of associations was broadly consistent with those observed in the primary analysis.

The climate index remained negatively associated with the reported incidence of mushroom poisoning (β = -0.073, 95% CI: -0.129 to -0.017). The education level index remained positively associated with incidence (β = 0.077, 95% CI: 0.020 to 0.135), while the urbanization rate remained negatively associated with incidence (β = -0.116, 95% CI: -0.202 to -0.029)and the lagged medical staff allocation index (β = 0.068, 95% CI: −0.010 to 0.145) were positively associated with incidence but did not reach statistical significance. No association was observed for hospital beds per 1,000 population (**Table S2**).

These findings indicate that the overall directions of association were generally consistent with those of the primary analysis, although the estimated effects of some lagged socioeconomic variables were attenuated and no longer statistically significant after introducing one-period lagged covariates.

**Table S2** Sensitivity analysis using one-period lagged explanatory variables

| Variable | β | Robust-SE | t | *P* | **95% CI** |
| --- | --- | --- | --- | --- | --- |
| Per capita GDP ( One-Period Lag) | 0.079 | 0.059 | 1.338 | 0.181 | (-0.037, 0.195) |
| Medical Staff Allocation Index ( One-Period Lag) | 0.068 | 0.040 | 1.702 | 0.089 | (-0.010, 0.145) |
| Climate Index | -0.073 | 0.028 | -2.568 | 0.010 | (-0.129, -0.017) |
| Education Level Index | 0.077 | 0.029 | 2.625 | 0.009 | (0.020, 0.135) |
| Urbanization Rate | -0.116 | 0.044 | -2.616 | 0.009 | (-0.202, -0.029) |
| Hospital Beds per 1,000 People | -0.024 | 0.015 | -1.594 | 0.111 | (-0.054, 0.006) |

2.2: Poisson/negative binomial count model with log population as offset

The surveillance data exhibited substantial overdispersion (dispersion parameter = 32.21), indicating that a Poisson model was inappropriate and supporting the use of a negative binomial specification.

Results from the random-effects negative binomial model were broadly consistent with those of the primary panel regression analysis, with GDP and educational attainment showing positive associations and climate index and urbanization showing negative associations with reported mushroom poisoning case incidence. Overall, the direction of all estimated associations remained generally consistent with those observed in the primary panel regression model, providing additional support for the primary findings despite differences in model specification.

**Table S3** Sensitivity analysis using a random-effects negative binomial regression model with log(population) as an offset

| Variable | β | SE | z | *P* | **95% CI** |
| --- | --- | --- | --- | --- | --- |
| Per capita GDP | 0.689 | 0.413 | 1.667 | 0.095 | (-0.120, 1.498) |
| Medical Staff Allocation Index | 0.398 | 0.347 | 1.147 | 0.252 | (-0.282, 1.078) |
| Climate Index | -0.449 | 0.182 | -2.464 | 0.014 | (-0.806, -0.092) |
| Education Level Index | 0.533 | 0.295 | 1.806 | 0.071 | (-0.045, 1.111) |
| Urbanization Rate | -0.824 | 0.457 | -1.805 | 0.071 | (-1.720, 0.072) |
| Hospital Beds per 1,000 People | -0.113 | 0.268 | -0.422 | 0.673 | (-0.638, 0.412) |

2.3 Sensitivity analysis additionally adjusting for calendar year

To further evaluate the potential influence of secular temporal trends, an additional sensitivity analysis was performed by including calendar year as a categorical variable in the random-effects panel regression model. The directions of the estimated associations remained unchanged, although the associations for the climate index and medical staff allocation index were attenuated after adjustment. None of the calendar-year indicators was statistically significant, suggesting that adjustment for calendar year did not materially alter the observed regional associations (**Table S4**).

**Table S4** Sensitivity analysis with additional adjustment for calendar year

| Variable | β | SE | t | P | 95% CI |
| --- | --- | --- | --- | --- | --- |
| Per capita GDP | 0.119 | 0.059 | 2.034 | 0.044 | (0.004, 0.234) |
| Medical Staff Allocation Index | 0.057 | 0.041 | 1.386 | 0.169 | (−0.024, 0.139) |
| Climate Index | -0.035 | 0.028 | -1.247 | 0.215 | (−0.090, 0.020) |
| Education Level Index | 0.070 | 0.032 | 2.159 | 0.033 | (0.006, 0.133) |
| Urbanization Rate | -0.153 | 0.055 | -2.769 | 0.006 | (−0.262, −0.045) |
| Hospital Beds per 1,000 People | -0.026 | 0.013 | -1.983 | 0.049 | (−0.052, 0.0003) |

Note: The model additionally adjusted for calendar year as a categorical variable. Regression coefficients for calendar-year indicators are omitted for brevity.

2.4 Temperature and precipitation as alternative climatic variables in the random-effects panel regression model

The association between temperature and reported mushroom poisoning incidence was not statistically significant, whereas precipitation remained negatively associated with incidence. Importantly, the directions of the associations for the remaining covariates were broadly consistent with those observed in the primary model, suggesting that the main findings were generally consistent across alternative climatic variable specifications (**Table S5**).

**Table S5**. Sensitivity analyses using temperature and precipitation as alternative climatic variables

| Variable | β | SE | z | P | 95% CI |
| --- | --- | --- | --- | --- | --- |
| **Model A. Temperature model** | | |  |  |  |
| Per capita GDP | 0.993 | 0.396 | 2.511 | 0.012 | (0.217, 1.769) |
| Medical Staff Allocation Index | 0.340 | 0.385 | 0.884 | 0.377 | (-0.415, 1.095) |
| Temperature | -0.145 | 0.239 | -0.609 | 0.543 | (-0.613, 0.323) |
| Education Level Index | 0.531 | 0.285 | 1.865 | 0.062 | (-0.028, 1.090) |
| Urbanization Rate | -1.060 | 0.443 | -2.395 | 0.017 | (-1.928, -0.192) |
| Hospital Beds per 1,000 People | -0.050 | 0.260 | -0.193 | 0.847 | (-0.560, 0.460) |
| **Model B. Precipitation model** | | |  |  |  |
| Per capita GDP | 0.719 | 0.403 | 1.785 | 0.074 | (-0.071, 1.509) |
| Medical Staff Allocation Index | 0.147 | 0.339 | 0.433 | 0.665 | (-0.517, 0.811) |
| Precipitation | -0.357 | 0.142 | -2.508 | 0.012 | (-0.635, -0.079) |
| Education Level Index | 0.579 | 0.293 | 1.974 | 0.048 | (0.005, 1.153) |
| Urbanization Rate | -0.790 | 0.465 | -1.701 | 0.089 | (-1.701, 0.121) |
| Hospital Beds per 1,000 People | -0.067 | 0.266 | -0.254 | 0.800 | (-0.588, 0.454) |
